# Pathogenesis of Cardiovascular and Metabolic Diseases: Are Fructose-Containing Sugars More Involved Than Other Dietary Calories?

**DOI:** 10.1007/s11906-016-0652-7

**Published:** 2016-04-28

**Authors:** Robin Rosset, Anna Surowska, Luc Tappy

**Affiliations:** Department of Physiology, Faculty of Biology and Medicine, University of Lausanne, Rue du Bugnon 7, CH-1005 Lausanne, Switzerland

**Keywords:** Fructose and cardiovascular disease, Sugars and cardiovascular disease, Metabolic disease and sugar, Visceral obesity, Uric acid, Sympathetic nervous system, Endothelial dysfunction

## Abstract

There is increasing concern that sugar consumption may be linked to the development of metabolic and cardiovascular diseases. There is indeed strong evidence that consumption of energy-dense sugary beverages and foods is associated with increased energy intake and body weight gain over time. It is further proposed that the fructose component of sugars may exert specific deleterious effects due to its propension to stimulate hepatic glucose production and de novo lipogenesis. Excess fructose and energy intake may be associated with visceral obesity, intrahepatic fat accumulation, and high fasting and postprandial blood triglyceride concentrations. Additional effects of fructose on blood uric acid and sympathetic nervous system activity have also been reported, but their link with metabolic and cardiovascular diseases remains hypothetical. There is growing evidence that fructose at physiologically consumed doses may exert important effects on kidney function. Whether this is related to the development of high blood pressure and cardiovascular diseases remains to be further assessed.

## Introduction

Obesity prevalence was low in Western societies and virtually absent in most South-American, African, and Asian countries at the beginning of the twentieth century. The number of individuals overweight or obese (i.e., body mass index ≥25 kg m^−2^) has however increased dramatically since then, to presently affect close to 40 % of the population worldwide [1]. This augmentation closely paralleled rises in the prevalence of coronary heart diseases, hypertension, and stroke [2].

The development of obesity results from a long-lasting imbalance between energy intake and energy expenditure and may occur following the excess consumption of any energy-containing nutrient. There has however been a growing concern that the deposition of body fat may be particularly favored by dietary sugars, that would at first sight appear at odds with the recommendations to consume at least five daily servings of fruits and vegetables. Yet, these adverse health effects were specifically assigned to “free” sugars, defined as “mono- and disaccharides added to foods by the manufacturer, cook, or consumer, plus sugars naturally present in honey, syrups, and fruit juices” [3]. Collectively, free sugars are estimated to represent ≈20 % of daily energy intake in westernized countries, mostly as mixtures of glucose and fructose [4].

## Why May Fructose Specifically Contribute to Cardiometabolic Risk?

Suspicions on fructose were initially raised by observational studies showing associations between cardiometabolic diseases and consumption of fructose-containing sugars (i.e., sucrose, high-fructose corn-syrup, and fruit juices) but not with lactose (a glucose-galactose dimer). These considerations were also confirmed by several pre-clinical and clinical studies globally showing that dietary fructose *can* induce several metabolic alterations bearing close similarity with the metabolic syndrome [5, 6]. The existence of direct causal link between fructose intake and the development of metabolic and cardiovascular diseases, however, remains also disputed [7, 8].

Unlike glucose and fatty acids that are key energy substrates and are metabolizable by most cells, fructose cannot be used as such and requires to be pre-processed in splanchnic organs [9]. In the liver, fructose undergoes a three-step intermediate metabolism (i.e., “fructolysis”) generating trioses-phosphates that join the glycolysis pathway. Since the main phosphorylating enzyme governing this sequence (fructokinase, converting fructose to fructose-1-phosphate) is unregulated, fructose intake has been proposed to lower hepatic ATP pools, thus stimulating hepatic nucleotide turnover and uric acid production [10]. Furthermore, fructose was considered to induce an overload of trioses phosphates that could either be converted to glucose, lactate, glycerol or fatty acids, or be directed to mitochondrial oxidation. The synthesized products, in turn, could be secreted in the circulation or furnish liver glycogen and triglycerides pools [11•]. As a remarkable result of this hepatocentric metabolism, fructose induces very little insulin secretion. These fructose-induced increases in hepatic glucose production and VLDL-TG secretion were proposed by several authors as being early markers of cardiometabolic diseases [5, 6]. In the next sections, we will discuss how fructose could specifically increase cardiovascular risk by promoting visceral and intrahepatic fat deposition, hypertriglyceridemia, and hypertension.

### Effects of Fructose on Visceral and Intrahepatic Fat Deposition

Results from many large and medium-sized cohort studies clearly indicate that the consumption of sugar, and more specifically of sugar-sweetened beverages, is related with weight gain over time [12•]. This association becomes weaker when adjusted for total energy intake, however, indicating that it is confounded by obesity. Of interest, some small-sized short-term intervention studies noticed that dietary fructose may be more specifically associated with visceral fat than glucose. The mechanisms responsible remain to be understood, yet, particularly since this effect was observed only in males in one of these trials [13]. If these data were to be replicated in other well-controlled larger studies, such an effect of fructose would indeed be a major health concern, given the strong association between visceral adipose tissue and both insulin resistance and cardiovascular risk.

**Fig. 1:**
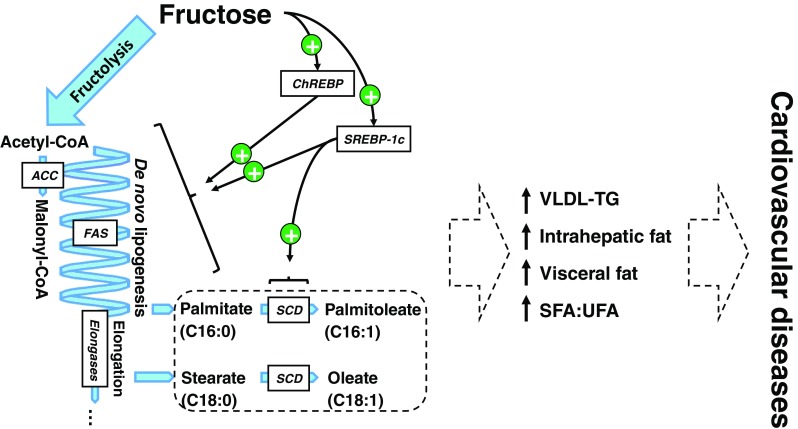
Potential roles of fructose-derived de novo lipogenesis on cardiovascular health. De novo lipogenesis, i.e., synthesis of long-chain fatty acids from acetyl-coA precursors, can occur following the pre-respiratory degradation of any nutrients. This pathway is particularly active during fructose overfeeding experiments, however, and the high lipogenic potential of fructose is due to (1) an unregulated synthesis of pyruvate and acetyl-CoA and (2) a stimulation of lipogenic transcription factors CHREBP and SREBP-1c by intracellular fructose metabolites. In turn, this stimulation of de novo lipogenesis may be associated with increased VLDL-triglyceride secretion and intrahepatic fat accumulation. The outcome of this process may also depend on the ratio of saturated to unsaturated fatty acids (SFA:UFA) in blood lipids, affected by dietary fat as well as by the relative activities of the enzymes involved in synthesis (fatty acid synthase), elongation (elongases), and desaturation (stearoyl-CoA desaturase) of newly generated fatty acids. Abbreviations: *ChREBP* carbohydrate-responsive element-binding protein, *SREBP-1c* sterol regulatory element-binding protein 1c, *CoA* coenzyme A, *ACC* acetyl-CoA carboxylase, *FAS* fatty acid synthase, *SCD* stearoyl-CoA desaturases, *VLDL-TG* triglycerides in very low-density lipoprotein fraction, *SFA:UFA* saturated-to-unsaturated fatty acids ratio

The relationship between cardiovascular and/or metabolic risk and visceral fat depots in general has been recently reconsidered in favor of a prevailing role of intrahepatic fat. It is indeed well established that the prevalence of non-alcoholic fatty liver disease is very high among subjects with abdominal obesity and insulin resistance. Furthermore, the study of subgroups of subjects with similar visceral fat mass but differing intrahepatic fat suggested that ectopic fat depots in the liver, rather than omental fat, may be directly linked with insulin resistance and cardiovascular risk [14]. Interestingly, it was observed that switching healthy volunteers from a low to a high-fructose diet significantly increased intrahepatic fat content within a few days [15]. This effect may be related with the conversion of a portion of fructose carbons into lipids through hepatic de novo lipogenesis. In this energetically costly anabolic pathway, mitochondrial citrate is first transported to the cytosol, then converted to acetyl-CoA and to malonyl-CoA. The latter, in turn, acts as a precursor for the iterative addition of two-carbon acetate chains to acetyl-CoA, resulting in the synthesis of palmitate (C16:0). Palmitate can be elongated to stearate (C18:0) and to fatty acids of longer carbon backbone by elongases, and both palmitate and stearate can be desaturated to palmitoleate (C16:1) and oleate (C18:1), respectively (Fig. 1).

The net effect of de novo lipogenesis on cardiovascular risk remains unclear and could largely depend on factors beyond the net mass of acetyl-CoA polymerized. The type of synthesized fatty acids may be particularly important, especially since high concentrations of palmitate have been well-described to promote atherogenesis. Alternatively, the activation of ChREBP and specifically of SREBP-1c could limit palmitate and stearate accumulation by promoting their desaturation into palmitoleate and oleate by SCD. Oleate, in turn, was found in vitro to protect against palmitate-induced lipotoxicity [16]. Consistent with a key role of saturated to unsaturated fatty acid ratio (SFA:UFA), SCD-deficient mice are highly insulin-sensitive and resistant to diet-induced obesity [17]. Similarly, the ratio of dietary fatty acids was also shown to critically affect carbohydrate-induced steatosis [18]. How these effects interplay in humans, and how they alter cardiovascular health could be investigated in Inuits, in which specific variants of desaturases and elongases were recently found [19].

Fructose has been amply documented to stimulate hepatic de novo lipogenesis more efficiently than glucose, and this was proposed to account for its role in non-alcoholic fatty liver disease. Recent short-term intervention studies, however, revealed that hypercaloric high-fructose and high-glucose diets both increased intrahepatic fat content to the same extent in healthy human volunteers [15, 20•]. The same was also observed for high-fat hypercaloric diet [21•]. In contrast, the consumption of weight-maintenance high-fructose diet led to a moderate stimulation of hepatic de novo lipogenesis [22••, 23•], but neither high-fructose nor high-glucose diets were associated with increased intrahepatic fat content [20•]. This suggests that, more than fructose per se, excess energy intake may be responsible for the development of hepatic steatosis.

### Effects of Fructose on Fasting and Postprandial Triglycerides

Many epidemiological studies indicated a strong association between sugar intake and blood triglyceride concentrations [24]. Fructose, in particular, was found in numerous short-term intervention studies to further increase fasting and postprandial triglycerides [13, 25] and may also alter cholesterol metabolism [26].

The mechanisms underlying fructose-induced hypertriglyceridemia remain controversial. On one hand [27], the conversion of fructose carbons into fatty acids by de novo lipogenesis may be responsible for both increased hepatic triglyceride synthesis and VLDL-triglyceride secretion, concomitantly enhancing circulating triglycerides appearance [28]. On the other hand, fructose was suggested to impair plasma clearance of triglyceride-rich lipoproteins, since co-ingested fructose enhanced the triglyceride response to a mixed meal [25]. In turn, both processes may vary according to population, fructose dose, and co-ingested nutrients.

Since high fasting and postprandial blood triglyceride are recognized to be independent risk factors for cardiovascular diseases, their augmentation following fructose intake is consistent with a role of fructose in cardiovascular risk. Accordingly, when fructose replaced starch in weight-maintaining controlled diets, it significantly raised blood triglyceride concentrations. Interestingly, these increases were larger and mainly observed when fructose was administered as part of hypercaloric diets, suggesting a modulation of this effect by energy balance. In turn, other factors may be involved, since moderate exercise normalized fasting and postprandial triglycerides concentrations, even in conditions of neutral energy balance [23•]. The available data, however, clearly indicate that fructose-induced hypertriglyceridemia generally remains within the normal range (i.e., under pathological thresholds), and whether this reflects a normal effect of fructose [29] or a true risk factor is still debated.

### Effects of Fructose on Blood Pressure and Hemodynamics

**Fig. 2:**
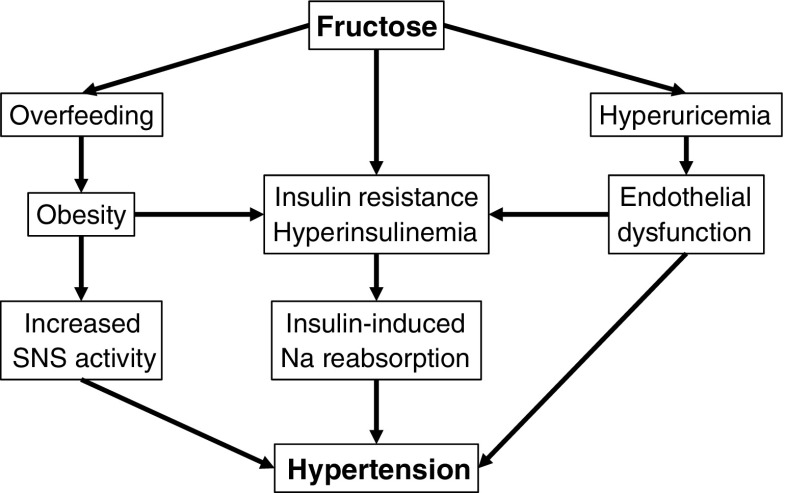
Potential mechanisms linking dietary fructose and hypertension. See text for detailed descriptions

**Fig. 3:**
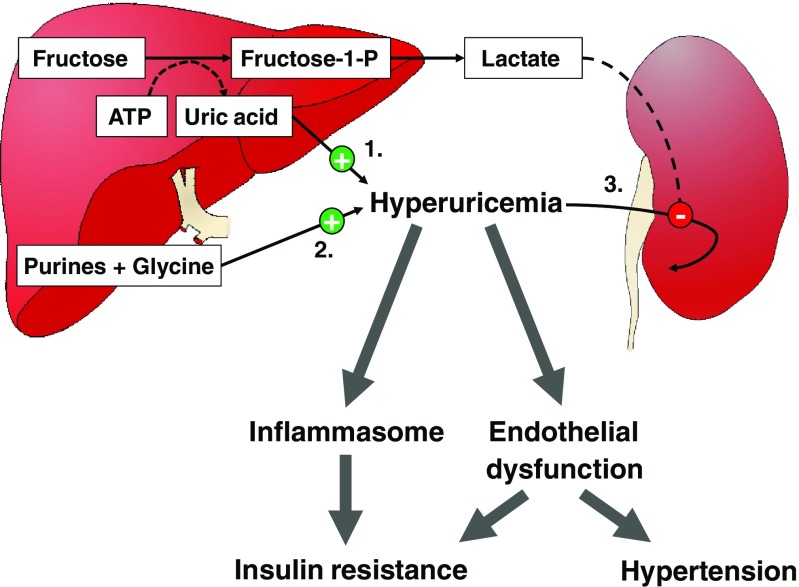
Potential deleterious effects of fructose-induced hyperuricemia. Fructose may increase plasma uric (i.e., hyperuricemia) acid through (1) transient intrahepatocellular energy depletion (due to ATP consumption by fructokinase following large i.v. fructose loads), (2) stimulation of endogenous uric acid synthesis from purine and glycine precursors (by unknown mechanisms), and (3) decreased uric acid excretion (by competition with lactate for secretion into urinary tubules). In turn, hyperuricemia may contribute to the metabolic effects of fructose by triggering inflammation through the NALP3 inflammasome or by impairing endothelial function, in turn promoting insulin resistance and hypertension. Hyperuricemia may also activate lipogenic enzymes (not shown). *ATP* adenosine triphosphate, *Fructose-1-P* fructose-1-phosphate
  Interestingly, it has recently been documented that uric acid may exert important regulatory effects in several physiological processes [52]. Hyperuricemia potently activates the NALP3 inflammasome (cryopyrin) [57] and may therefore trigger a low-grade chronic inflammation secondarily responsible the development of insulin resistance [58]. An increase in uric acid in the liver may stimulate fructose metabolism [59•] and lipogenic enzymes [60•] and may thus contribute to further enhance de novo lipogenesis. High uric acid concentration may also impair normal endothelial function and may contribute to the development of hypertension or to that of insulin resistance by preventing insulin-induced vasodilation [61]. The latter mechanisms have been reported in rodent models of high-fructose diet, while human observations were more mitigated. One study reported that middle-aged, hypertensive, obese high-fructose consumers had significantly reduced blood pressure when their blood uric acid concentrations were lowered with administration of allopurinol [32]. However, this was not associated with any improvement of markers of insulin resistance such as glucose and insulin concentration nor of dyslipidemia.
- Kidneys Dysfunction
  The effects of dietary fructose on blood pressure remain altogether highly controversial. Fructose is associated with the development of hypertension in some, but not all rat models fed a high-fructose diet. The explanation responsible between studies differences remains unknown, but other co-variables are likely to be involved. Some investigators have suggested that hypertension occurring in some high-fructose fed animals may be due to some experimental artifacts such as diet copper [62] or magnesium [63] deficiencies.
  Of more importance, there is growing evidence that fructose enhances the effects of a high-salt diet on blood pressure [64••]. This may result from multiple effects of fructose on renal handling of sodium and other ions. Fructose has indeed been shown to increase the reabsorption of sodium in the proximal tubule through various mechanisms, including enhanced bicarbonate reabsorption and increased sodium-hydrogen exchangers [65].
  Major regulatory steps of sodium and water metabolism linked to blood pressure control take place in the distal renal tubule. Of interest, filtered glucose is normally completely reabsorbed from the primary urine in the proximal tubule and does not reach the distal renal tubule apart in overt diabetes mellitus. Chronic hyperglycemia and glycosuria in diabetes mellitus are associated with increased kidneys size and the progressive development of renal and cardiovascular dysfunction. Compared to glucose, blood fructose concentrations remain most of the time very low and can only transiently reach 0.5–0.8 mmol L^−1^ following a fructose-containing meal. Under such conditions, however, fructose can be filtered by the kidneys and appears in primary urine. In contrast to glucose, which can be efficiently reabsorbed in the proximal tubule through an active sodium-glucose co-transport, fructose is only removed from the proximal tubule by passive diffusion. It seems thus conceivable that dietary fructose can reach the distal parts of the nephron, where its effects on renal function remain to be evaluated [66].
  Epidemiological studies show an association between sugar [24] and fructose [30] intake and the development of hypertension or cardiovascular diseases, yet obesity is an important confounder in these analyses. There is strong evidence that a high sugar intake is frequently encountered in obese subjects, and part of these associations may therefore be related to excess body weight or insulin resistance. In support of this hypothesis, insulin-resistant subjects have chronically elevated hyperinsulinemia, which has been shown to increase renal sodium reabsorption [67]. There is also evidence that endothelial function is frequently impaired in insulin resistant subjects, possibly due to lipid-mediated endothelial toxicity. Such endothelial dysfunction have minimal effects on resting arterial pressure, but can be associated with excessive blood pressure rise in response to external stimuli such as stress [45, 46].

Recent epidemiological data from large cohort studies show a strong positive relationship between sugars [24] or fructose [30] intake and hypertension. Both associations, interestingly, were markedly attenuated when adjusted for body weight, suggesting that obesity was an important confounder. Few short-term intervention studies reported the effects of altering dietary fructose content on blood pressure. Although most of them did not report any significant effect of on blood pressure [13, 31], one study documented a 6 % increase in ambulatory blood pressure in middle-aged men with the metabolic syndrome [32]. Several mechanisms may potentially account for hypertensive effects of fructose (Fig. 2) [33]:

- Sympathetic Nervous System (SNS) Activation
  Carbohydrate ingestion and intravenous infusion of glucose both increase sympathetic nervous system activity, the effects of which are highly complex and only partly elucidated. SNS activity was suspected to be protective against obesity in some individuals through allowing energy wasting. Consequently, it has been intensively investigated in studies measuring the concentrations and turnover of norepinephrine [34], as well as organ-specific microneurography [35]. These reports, however, resulted in little support of an anti-obesogenic effect, but demonstrated that SNS activation was much more complexly orchestrated than previously thought, and was differentially regulated in various organs.
  The functional significance of food-induced sympathetic activation remains largely hypothetical. Glucose and insulin infusion both increase markers of SNS activity, and at the same time, stimulate resting energy expenditure. Since this effect can be partially inhibited by administration of beta-adrenergic antagonists [36], SNS was considered to be involved in adaptive thermogenesis. Such SNS-mediated heat generation was initially proposed to mainly occur in brown adipocytes in rodents [34] and in skeletal muscle in humans [37]. This concept, however, may need to be revised following demonstrations of brown adipose tissue existence in healthy human adults [38]. These specialized cells, alike recently discovered “beige” adipocytes [39], can generate heat by uncoupling mitochondrial respiration from ATP synthesis and may be active during stressful situations such as cold exposure or exercise [40]. Whether this is related to food-induced sympathetic activation and regulation of body temperature remains speculative, however.
  SNS activation varies according to the type of carbohydrate consumed and to the organ considered. Glucose administration, either intravenous or oral, stimulates muscle sympathetic nerve activity [41]. This effect of glucose appears to be primarily mediated by insulin and is coupled with insulin-induced NO-ergic vasodilation of skeletal muscle arterioles [42, 43]. Interestingly, fructose, as glucose, increases postprandial energy expenditure, and this effect could be lowered by about 30 % using beta-adrenergic antagonists [44]. However, glucose activates muscle sympathetic nerve activity while fructose does not, suggesting that stimulation of adaptive thermogenesis by fructose is independent of insulin secretion and does not involve muscle sympathetic nerve activation.
  The effects of the sympathetic nervous system on cardiovascular and hemodynamic functions are highly complex and only partially elucidated. They involve the coordinated activation of several subsets of sympathetic nervous fibers, as was illustrated by studies on mental stress [45, 46]. Mental stress can be elicited in animals by forced immobilization and in humans by complex experimental paradigms reproducing what happens in a prey when encountering a predator. Activation of the sympathetic nervous system altogether with stimulation of glucocorticoids and catecholamines from the adrenal medulla then elicits a set of coordinated hemodynamic and metabolic responses to allow for “fight or flight” responses (i.e. aiming to immediately optimize muscle functions to outrun the predator or eventually to fight for life). These include increases in cardiac output and ventilation in order to raise systemic blood flow and oxygenation. Simultaneously, circulating substrates are raised following the stimulation of hepatic glucose output and adipose tissue lipolysis by glucocorticoids and sympathetic mediators. Finally, muscle sympathetic nerve activation occurs, inducing a local vasodilation that favors as a whole an increased delivery of oxygen and energy substrates to skeletal muscle, ensuring optimal immediate response.
  Food intake also elicits a set of integrated hemodynamic adaptations to favor blood perfusion in digestive organs at the expense of most other tissues excepting the brain. SNS activation, again, is instrumental in many of these effects, notably by increasing both heart rate and contraction force, thus raising cardiac output [47]. Even if digestive organs vasodilation is essentially produced by local autoregulatory mechanisms, episodes of severe postprandial systemic hypotension in individuals with autonomic neuropathy indicate that SNS activation is still required to maintain physiological blood pressure [48]. This is mainly attained by alpha-adrenergic vasoconstriction of arterioles in non-digestive vascular beds in the kidneys and the skin. Surprisingly, ingestion of a meal is associated with sympathetic nerve activation altogether with arteriolar vasodilation in skeletal muscle. This effect appears mediated by insulin-induced release of nitric oxide from muscle endothelium, possibly triggered by activation of sympathetic NO-ergic fibers. This unexpected vasodilation in skeletal muscle induced by meal ingestion has also now been recognized to contribute to insulin sensitivity by facilitating the delivery of both glucose and insulin to skeletal muscle.
  Acute administration of a fructose drink, but not of glucose, produces a small and transient increase in systolic and diastolic blood pressure in healthy humans [49]. This may indicate that fructose activates vasoconstrictive sympathetic fibers in some non-digestive organs to a larger extent than glucose and hence may favor the development of hypertension is some individuals. This effect was however observed with administration of pure fructose only, but disappeared when glucose and fructose were co-ingested due to glucose-induced, insulin-dependent muscle vasodilation [50]. This therefore suggests that ingestion of fructose-containing sweeteners such as sucrose, honey, and high-fructose corn syrup is unlikely to produce large acute increases in blood pressure.
- Hyperuricemia
  Intravenous infusion of large fructose loads has been recognized since several decades to result in several metabolic perturbations including hyperlactatemia, hypoglycemia, and hyperuricemia [10]. The same effects can be observed when subjects with hereditary fructose intolerance ingest even small amounts of fructose or sorbitol. This condition is due to an inherited deficiency of aldolase B, with the consequence that fructose is phosphorylated to fructose-1-P in fructokinase-expressing cells, a reaction which is associated with ATP consumption [51]. The absence of aldolase B thereafter impairs further metabolism of fructose-1-P and thus prevents ATP regeneration. This leads to ATP depletion and energy crisis in fructokinase-expressing cells such as hepatocytes, in which this impairs glucose production, thus leading to hypoglycemia. In the kidneys, it leads to proximal tubular dysfunction and acidosis. In addition, ATP depletion stimulates the degradation of ADP to AMP and inositol, thus increasing uric acid production [52]. It is likely that, in normal subjects, the i.v. administration of large amounts of fructose leads to a rapid phosphorylation of fructose to fructose-1-phosphate, which outpaces temporarily the ability of aldolase B to clear fructose-1-phosphate. It results that i.v. administration of fructose is associated with an acute increase in blood uric acid and can occasionally cause acute metabolic defects similar to those encountered in hereditary fructose intolerance.
  Administration of oral physiological fructose loads is occasionally associated with moderate postprandial increases in blood uric acid concentration, the latter effect being however inconstant [53]. In contrast, an increase in dietary fructose intake is associated with moderate, yet highly significant elevations in fasting uric acid concentrations [53]. This effect is consistently observed in hypercaloric, high-fructose diets when fructose iso-energetically replaces starch or fat. This long-term moderate hyperuricemia is unlikely to be explained by ATP depletion, since it is not associated with the landmark effects of energy crisis in liver cells (i.e., hypoglycemia) or in renal tubules (i.e., proximal tubule dysfunction and renal acidosis). Instead, most subjects with hyperuricemia secondary to ingestion of a high-fructose diet present evidence of increased hepatic glucose production and de novo lipogenesis [54], two energy-requiring metabolic processes unlikely to be active in ATP-depleted cells. In turn, consumption of a high-fructose diet is almost invariably associated with an increase in blood lactate concentrations [46], but this merely reflects a physiological conversion of fructose into a more ubiquitously usable energy substrate, rather than a state of acidosis. Early isotope studies indicated that fructose may stimulate the endogenous synthesis of uric acid [55], but the exact mechanism and functional significance of this remains unknown. In turn, the study of kidneys transporter proteins has dramatically progressed over the past decades and provides additional putative mechanisms. Uric acid and lactate share the same transporter in proximal tubule cells, URAT1, and the high blood lactate concentrations observed after fructose intake may compete with uric acid filtration and decrease uric acid renal clearance (Fig. 3) [56].

## Conclusion

In a well-balanced analysis, Casazza and colleagues noticed that “passionate interests, the human tendency to seek explanations for observed phenomena, and everyday experience appear to contribute to strong convictions about obesity” [68]. In the context of fructose research, these words of caution sound particularly pertinent, and many mechanisms still need to be understood before reaching definitive conclusions. There is indeed a strong evidence that a high sugar intake is present in many obese patients, and that can be associated with the development of non-communicable diseases. In our opinion, this is mostly explained by the hedonic properties of sugary foods, which tend to favor the overconsumption of highly palatable energy-dense foods when available. Compared to other nutrients such as glucose or fat, fructose is first processed in splanchnic organs and then released as glucose, lactate, or VLDL-TG into the systemic circulation. This may contribute to the development of and hepatic insulin resistance during chronic excessive intake of both total energy and sugar. Additional effects of fructose on blood uric acid and sympathetic nervous system activity have also been reported, but their link with metabolic and cardiovascular diseases remains hypothetical. Interestingly, there is growing evidence that more fructose may escape first pass hepatic liver metabolism than previously thought, and that systemic low fructose concentrations may exert important effects on kidney function. Whether this is related to the development of high blood pressure and cardiovascular diseases remains to be further assessed.

## Abbreviations

ATP, ADP, AMP: Adenosine tri-, di- and monophosphate
ChREBP: Carbohydrate-responsive element-binding protein
SREBP-1c: Sterol-regulatory element-binding protein 1c
CoA: Coenzyme A
ACC: Acetyl-CoA carboxylase
FAS: Fatty acid synthase
SCD: Stearoyl-CoA desaturases
HDL: High-density lipoproteins
VLDL: Very low-density lipoproteins
SNS: Sympathetic nervous system
NO: Nitric oxide
